# Plants and Phytoplasmas: When Bacteria Modify Plants

**DOI:** 10.3390/plants11111425

**Published:** 2022-05-27

**Authors:** Assunta Bertaccini

**Affiliations:** Department of Agricultural and Food Sciences, Alma Mater Studiorum—University of Bologna, 40127 Bologna, Italy; assunta.bertaccini@unibo.it

**Keywords:** plant diseases, bacterium, symptoms, pathogenicity, molecular classification

## Abstract

Plant pathogen presence is very dangerous for agricultural ecosystems and causes huge economic losses. Phytoplasmas are insect-transmitted wall-less bacteria living in plants, only in the phloem tissues and in the emolymph of their insect vectors. They are able to manipulate several metabolic pathways of their hosts, very often without impairing their life. The molecular diversity described (49 ‘*Candidatus* Phytoplasma’ species and about 300 ribosomal subgroups) is only in some cases related to their associated symptomatology. As for the other plant pathogens, it is necessary to verify their identity and recognize the symptoms associated with their presence to appropriately manage the diseases. However, the never-ending mechanism of patho-adaptation and the copresence of other pathogens makes this management difficult. Reducing the huge impact of phytoplasma-associated diseases in all the main crops and wild species is, however, relevant, in order to reduce their effects that are jeopardizing plant biodiversity.

## 1. Introduction

**Figure 1:**
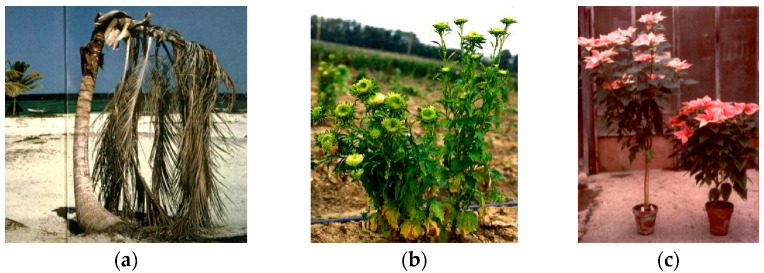
Coconut (**a**), aster (**b**), and poinsettia (**c**) infected by phytoplasmas.

Plant pathogen presence is usually known as a very dangerous component of agricultural ecosystems and is associated with huge economic losses. The world history was also often shaped by dangerous plant epidemics or pandemics such as the wheat rust that was among the main causes of the Roman empire failure, the potato late blight by *Phythophthora infestans* producing the Irish migration to America due to the famine, and the coffee rust obliging to stop the coffee cultivation is several areas, mainly in islands. Recently plant pathogenic bacteria have played an important role in reducing kiwi cultivation, due to the canker by *Pseudomonas syringae* pv. *actinidiae* [1], and citrus, through the greening (‘*Candidatus* Liberibacter’ species) [2] diseases. Moreover, there are bacteria hosted by plants and insects that are both associated with severe epidemic or with useful changes in plant behavior. While their presence in apple trees causes severe losses in production and kills millions of coconut palm trees in the Caribbean, the presence of a poinsettia branching bacterium is allowing its commercial production as pot plant (Figure 1).

Phytoplasmas are insect-transmitted wall-less bacteria provisionally classified to be the ‘*Candidatus* Phytoplasma’ species [3,4]. They live only in the plant phloem tissues and in the emolymph of their insect vectors, especially concentrated in the salivary glands. Their relationship with both plants and insects is very intimate and they are able to manipulate several metabolic pathways, very often without impairing the host’s life [5]. 

## 2. Phytoplasma Discovery

**Figure 2:**
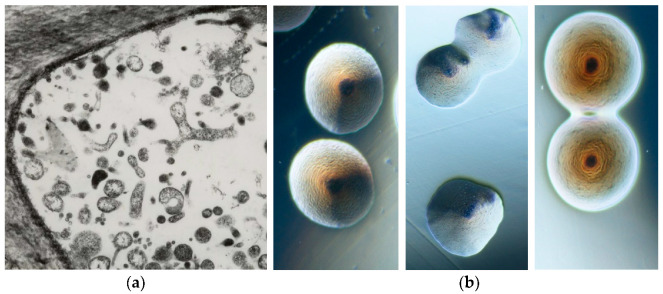
Transmission electron microscopy picture of a thin section in the phloematic tissue of a phytoplasma infected gladiolus plant showing the presence of strong pleomorphism (×8000) (**a**). Tree morphotypes of colonies containing phytoplasmas under binocular microscope (×40) (**b**).

The phytoplasma presence in plants historically dates back about 1000 years, when special tree peonies exhibiting green flowers were given to the Chinese court during the Song dynasty (900 BC) as the most precious and beautiful flower of the empire. However, scientific records of phytoplasma-associated plant diseases started when, in 1967, mulberry dwarf, rice yellow dwarf, and sweet potato witches’ broom, long considered to be caused by viruses, using electron microscopy, were found to be colonized by small pleomorphic bodies (80–800 nm in diameter) resembling mycoplasmas (bacterial pathogens of humans and animals) and were named mycoplasma-like organisms (MLOs) [6]. Their discovery stimulated worldwide investigation and numerous plant diseases were associated with the consistent presence of MLOs. These bacteria were long considered unculturable, but about 10 years ago, colonies containing molecularly different phytoplasmas began to be obtained in artificial media from different infected plant species (Figure 2) [7,8,9,10,11].

## 3. Phytoplasma Classification

The ‘*Candidatus* Phytoplasma’ genus provisional classification is highly relevant due to its application in epidemiological and ecological studies, mainly aimed at keeping the severe phytoplasma plant diseases under control worldwide. The updated proposed guidelines accommodate those ‘*Ca*. Phytoplasma’ species strains sharing > 98.65% sequence identity of their full or nearly full 16S rRNA gene sequences, obtained with at least 2-fold coverage of the sequence, compared with those of the reference strain of such species [4]. The officially published ‘*Candidatus* Phytoplasma’ species are 49; however, they do not cover all the relevant biodiversity, especially in reference to differential geographic distribution and/or host species. Therefore, the differentiation in ribosomal groups and subgroups [12] is still valuable and should be used to be able to work on their epidemiology and prevention in the different areas of the world. The main distribution of strains is tightly related to the geographic areas and to the dissemination performed by propagation materials, such as cuttings and seeds, that are also infected, even if only in low percentages (1–3%).

## 4. Relationship between Phytoplasma Symptomatology and Classification

Together with the study on the diseases associated with the presence of these bacteria, the first step was to give them a name according to the diverse disease in which the association was detected with specific phytoplasmas. Today, 30 years after this exercise started it appears clear that the molecular diversity described in phytoplasmas (49 ‘*Candidatus* Phytoplasma’ species and about 200 ribosomal subgroups) using the 16S ribosomal gene as basic standard is only in some cases related to a differential symptomatology. Identical symptoms are associated with different phytoplasmas and vice versa. Moreover, phytoplasmas associated with decline symptoms in some species could be associated with phyllody/virescence in others, such as ‘*Ca*. P. solani’ infecting potatoes, tomatoes, and grapevine. Therefore, contrary to the other plant pathogens it is necessary to verify the pathogen identity by molecular tools on a case-by-case basis; however, at the same time, it is of utmost importance to also recognize the symptoms associated with the presence of the phytoplasmas in order to appropriately manage the disease. This review of the main symptoms and several associated phytoplasmas worldwide is aimed at helping the recognition of the presence of these bacteria in plants, further clarifying their relationship with the host plants. This feature is, however, not stable over time also in the same plant species, considering the never-ending mechanism of patho-adaption that is part of life also in microorganisms; pathogens are special microorganisms that are simply looking for new ecological niches to ensure their survival and do not aim to destroy or kill the hosts.

### 4.1. Shoot Proliferation and Witches’ Broom

**Figure 3:**
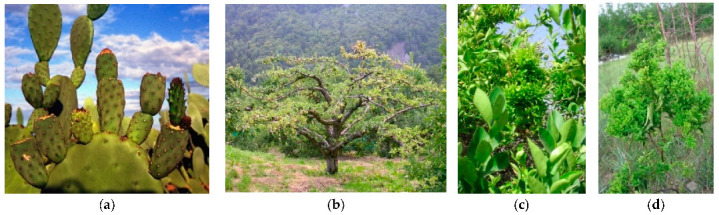
Cactus pear (*Opuntia ficus-indica*) proliferation (**a**), apple proliferation (**b**), citrus witches’ broom (**c**), and jujube witches’ broom (**d**) are associated with the presence of phytoplasmas in diverse areas of the world.

Diseases with symptoms of witches’ broom can be caused by basidiomycetes but could also be associated with the presence of phytoplasmas. In both cases they are economically important in a number of crop plant species, including the cocoa tree, jujube, citrus, and apple and timber trees, such as poplar, *Melia azedarach*, and paulownia (Figure 3). Among woody species, this malformation is almost always associated with the presence of specific phytoplasmas, such as in apple (‘*Ca*. P. mali), lime (‘*Ca*. P. aurantifolia’), lilac (‘*Ca*. P. fraxini’), paulownia (‘*Ca*. P. asteris’), almond (‘*Ca*. P. phoenicium’), *Juniperus* (16SrIX-E), walnut (16SrIII-G), *Balanites triflora* (‘Ca. P. balanites’), spartium (‘*Ca*. P. spartii’), black alder (16SrX-E), hibiscus (‘*Ca*. P. brasiliense’), *Guazuma* spp. (16SrXV-B), chestnut (‘*Ca*. P. castaneae’), *Cassia italica* (‘*Ca*. P. omanense’), and salt cedar (‘*Ca*. P. tamaricis’). In herbaceous host plants, the presence of witches’ broom was reported in diverse species, some of them as hosts of new phytoplasma strains (Table 1), such as strawberry, peanut, cactus, tabebuja, tomatillo, chayote, black raspberry, erigeron, alfalfa, and pigeon pea.

The excessive shoot proliferation results in poor or no fruit production and severely reduces the cultivation of some of these crops. Citrus in the Arabian Peninsula, jujube in China, and apple proliferation in Europe are some of the most severe cases that greatly reduce the possibility to produce and commercialize popular fruits. This modification is due to the loss of apical dominance of the shoots linked to disorders in the hormone balance.

### 4.2. Stunting and Little Leaf

**Figure 4:**
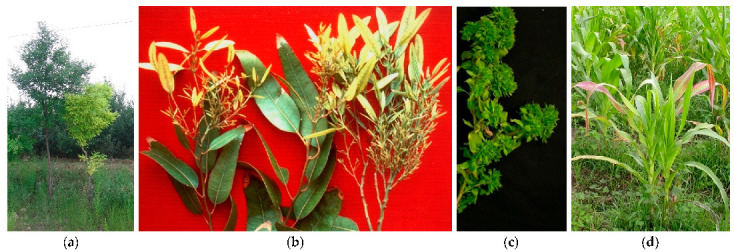
*Sophora japonica* stunting and yellows (**a**); *Eucalyptus* little leaf (**b**), periwinkle little leaf (**c**), and corn stunting (**d**).

Stunting in plants could be due to virus or phytoplasma presence; however, it must also be verified that glyphosate or similar pesticides were not applied in the area in which these malformations are present in plants in the past years, since this can produce indistinguishable symptoms (Figure 4). The presence of phytoplasmas is reported in several plant species enclosing small fruits, vegetables, corn, and soybean; in some cases, these bacteria were associated with the presence of little leaf or stunting also in trees, such as cherry, eucalyptus, and *Sophora japonica* [49] (Table 2). In strawberries the case *Fragaria multicipita* was discovered to be not a true species, but just a cloned phytoplasma-infected genotype [15]. The hormone imbalance, according with the diverse infected species, is usually present and the transportation of starch and other metabolites for the appropriate development is very often impaired.

### 4.3. Phyllody and Virescence

**Figure 5:**
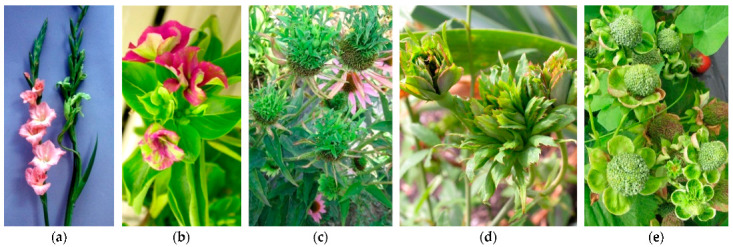
Virescence in gladiolus (**a**) and in periwinkle (**b**); phyllody in echinaea (**c**), rose (**d**), and strawberry (**e**). The rose flowers are showing virescence and phyllody due to genetics, rather than the phytoplasma presence in all the others.

The transformation of different plant organs into leaves is a very relevant symptom among those associated with phytoplasma presence and is known as phyllody; this type of malformation could also be due to the application of pesticides based on hormone-like molecules. The virescence is the change of the color of flowers to green, which is due to phytoplasma presence, but in some cases the diagnostics can be tricked by the existence of flowers that are green and the presence of genetic factors modifying the anthocian distribution in the plant, as can be seen in a Chinese variety of rose and in some special clones of periwinkle (Figure 5). The most relevant phytoplasma-associated diseases are reported in flowering species for commercialization; however, virescence is also present in horticultural and seed crops, such as tomatoes, cabbages, strawberries, and clover, among several other species (Table 3).

### 4.4. Yellowing and Decline

**Figure 6:**
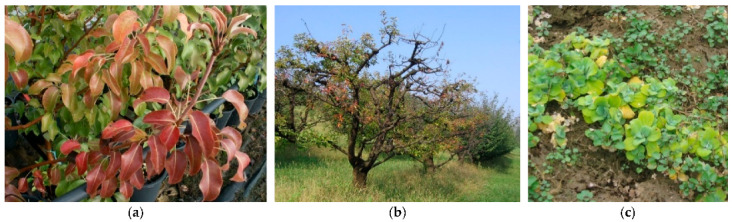
Reddening in pear decline (**a**), yellowing in plum (**b**) and watercress (**c**).

**Figure 7:**
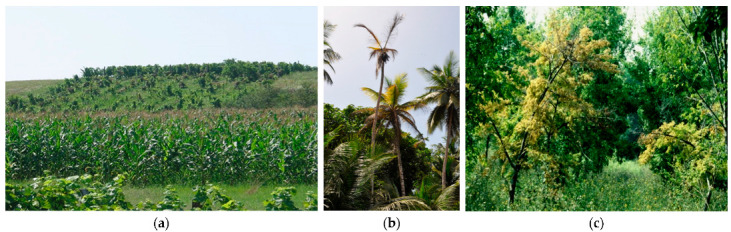
Grapevine yellows (**a**), coconut lethal yellowing (**b**), and elm yellows (**c**) associated with the presence of diverse phytoplasmas.

One of the main symptoms associated with the presence of phytoplasmas is the yellowing, in several cases these bacteria are also known as agents of yellows diseases. Generally, the yellowing of the aerial portions of the plant is complemented by a general decline that led to a huge, and in several cases complete, loss of production (Figure 6 and Figure 7). However, these symptoms can also be due to lack of nutrients, poor fertilization, and the presence of other pathogens infecting the root apparatus. The presence of phytoplasmas in plants exhibiting decline and yellowing must be considered together with these other factors in complex syndromes. The phytoplasmas associated with these symptoms are detected in some of the most economically relevant woody species, such as grapevine, fruit trees, and palms (especially coconut and other species for nut production) (Table 4). The metabolic basis for these symptoms is still very poorly understood, but the excessive consumption of sugar and the lack of its mobilization to the sink organs are involved.

### 4.5. White Leaf

**Figure 8:**
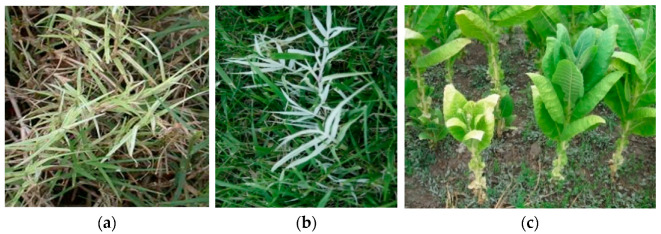
Bermudagrass white leaf in *Cynodon dactilon* (**a**,**b**) and yellow and stunting in tobacco (**c**).

The white leaf symptomatology (Figure 8) is limited to a small range of species, monocotyledonous, and is reported to be associated with phytoplasma presence only in Asia and Europe (Table 5). The main economically relevant disease is the sugarcane white leaf that is severely infecting this crop in all Asian countries. The presence of diverse phytoplasmas is associated in sugarcane also with other symptoms, such as yellow leaf and grassy shoots. These diseases are insect- and cutting-transmitted and in some cases also transovarially [95]. The lack of chlorophyl in the leaves is the main modification, which is often accompanied by a strong shortening of the cycle span and early drying; inappropriate photosynthesis is the mechanism involved in this modification of plants.

### 4.6. Purple Top and Other Malformations

**Figure 9:**
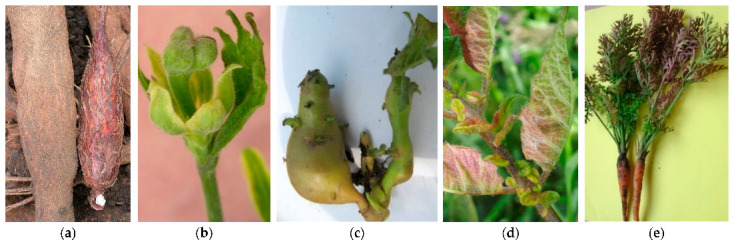
Cassava frog skin (**a**), tomato (**b**) and potato “stolbur” (**c**), potato purple top (**d**), and carrot reddening (**e**).

The presence of phytoplasmas in the sieve tube also interferes with the composition of the phloem sap and is associated with hormone imbalance; therefore, several diverse malformations in roots, flowers, tubers, and leaves can be observed in infected plants (Figure 9). Phytoplasmas that induce these malformations infect mainly herbaceous crops (Table 6).

## 5. Phytoplasma Genomics

Unlike common bacteria and many other organisms, including animals and plants, mycoplasmas use the UGA stop codon as a tryptophan-encoding codon; moreover, a gene encoding peptide chain release factor 2 that recognizes UGA as a termination codon is present in the phytoplasma genome [116]. The first complete genome sequence of 860,631 bp of the mutant OY-M that was reported in 2004 with a GC content of 28% [117]. Gene annotation analysis revealed that although the genome encoded basic cellular functions including DNA replication, transcription, translation, and protein translocation, the genes required for amino acid and fatty acid biosynthesis, the tricarboxylic acid cycle, and electron transport/oxidative phosphorylation were not present. Although metabolic genes were few in number, the OY-M genome contained many transporter genes. The phytoplasma genome is also rich in repeat regions with duplicated genes and transposon-like elements called potential mobile units; these features are similar to genes and organized in a conservative manner and are thought to play roles in the regulation of gene expression and serve as drivers for phytoplasma interaction with insects and plants [13,118,119,120].

## 6. Mechanisms to Infect Plants and Insects

The longtime search for pathogenicity factors still did not elucidate this important aspect and very little clarification is available about a basic question, which is: are phytoplasmas always pathogenic? The finding of several cases of phytoplasma presence in asymptomatic plants do not allow to answer to this question yet. Phytoplasmas are spread between plants by phloem-feeding insects, such as leafhoppers, planthoppers, and psyllids [121]. Due to their wide range of plant hosts, phytoplasmas are often detected in various crops and wild plants [122]. Because phytoplasmas are transmitted transovarially in several cases [123], the presence or absence of insect hosts is a critical determinant of their survival in the natural environment. When phytoplasmas invade insects, their extracellular membrane proteins play important roles for host interactions. Notably, antigenic membrane protein (AMP), a representative of phytoplasma membrane proteins that is predominantly detected on the phytoplasma cell surface, was found to form a complex with host microfilaments determining whether an insect can transmit a phytoplasma [124,125,126,127]. Furthermore, microarray and gene expression patterns analyses revealed that phytoplasmas dramatically alter the expression of approximately one-third of their genes using transcription factors to establish host switching between plants and insects [128].

## 7. Genetic Factors Determining Symptom Development

Some of the molecular mechanisms by which phytoplasmas induce their most typical symptoms were elucidated. Comparing the genome sequences of OY-W and OY-M revealed the duplication of glycolytic gene clusters in the OY-W genome. It has been suggested that this difference is responsible for the high consumption of carbon sources, resulting in high growth rates and severe symptoms, such as yellowing, dwarfism, and decline, at least in the case of the OY-W phytoplasma strain [129]. Furthermore, the mechanisms of purple top symptoms have been revealed. Phytoplasma infection activates the anthocyanin biosynthetic pathway. The increased accumulation of anthocyanin not only changes the color of the leaves to purple, but also acts as an antioxidant that protects plant cells from damage caused by reactive oxygen species, which results in leaf cell death [130].

A comprehensive search for pathogenicity-related genes, in which phytoplasma genes encoding secreted proteins were expressed. In 2009, the first phytoplasma effector protein, TENGU, a secreted peptide of 38 amino acids, was identified as an inducer of witches’ broom [131]. It is conserved among various phytoplasma strains. Following secretion from the phytoplasma cell, TENGU is cleaved in planta to a peptide of 12 amino acids, which is then transported to the shoot apical meristem, wherein it inhibits the signaling pathway of the plant hormone auxin and induces witches’ broom symptoms [132]. TENGU also induces the sterility of male and female flowers by inhibiting the signaling pathway of jasmonic acid (JA) [133]. The reduction in endogenous JA levels is thought to contribute to attracting insect vectors. Similarly, another secreted protein, SAP11, downregulates JA synthesis and increases the fecundity of insect vectors. 

In phytoplasma-infected plants, phyllody often affects sepals, and abnormal expression patterns of MTFs genes were found in all floral organs except stamens in phytoplasma-infected petunias [134,135]. Recently, SAP54 and PHYL1 were found to be homologous proteins that induce phyllody in the floral organs of *Arabidopsis thaliana.* The proteins interact with and then degrade A- and E- class MTFs via the ubiquitin–proteasome pathway and are genetically and functionally conserved among phytoplasma strains and species. Therefore, the phyllody-inducing gene/protein (phyllogen) family was demonstrated to induce flower phyllody and related malformations (virescence and proliferation). Phyllogens induce flower phyllody in various angiosperms and MTF degradation in non-flowering plants. These molecules induce virescence, phyllody, and proliferation symptoms, indicating that these flower symptoms are not independent symptoms induced by distinct effectors but a series of gradually changing phenotypes. Flower virescence can be considered just a mild form of phyllody, and the loss of flower meristem determinacy can be considered a severe form of phyllody [136,137,138,139].

Why do phytoplasmas induce symptoms accompanied by unique morphological changes, such as witches’ broom and phyllody? Both symptoms increase the prevalence of short branches and small young leaves, which are preferred by sap-feeding insects. Furthermore, phyllody flowers remain green even when healthy flowers wither. These features are likely to enhance the attraction of insect vectors and thus the spread of phytoplasmas. Such manipulations of the morphology of host plants appear to be a common strategy for the survival of phytoplasmas.

## 8. Management

Because phytoplasmas are difficult to culture, electron microscopy observation using ultrathin sections of sieve elements and plant recovery after tetracycline treatment were the only diagnostic methods available when phytoplasmas were discovered. Subsequently, several DNA-based technologies to detect phytoplasmas have been developed and applied routinely [140] to detect and correctly identify the phytoplasmas present in diseased crops and devise appropriate management strategies [141]. Although treatment using tetracycline-class antibiotics suppresses phytoplasma multiplication in infected plants cultured *in vitro*, high concentrations of antibiotics damage the plant tissues [142,143,144,145]. Recently, a comprehensive screening of 40 antibiotics showed that phytoplasmas were eliminated from infected plants not only by the application of tetracycline but also by using rifampicin. Diverse alternative and more sustainable methods were tested and are under trial for practical application; however, the production of phytoplasma-free nursery stocks is still the basis of a friendly and sustainable management, since curing plants is time- and money-consuming, considering that this pathogens are insect- and seed-transmitted. Methods to eliminate phytoplasmas from crops using diverse molecules and resistance inducers showed increased plant performances but not pathogen elimination, and in many cases the scaling up of these systems has not yet been exploited.

## 9. Concluding Remarks

In the last quarter century, although there have been many barriers to the study of phytoplasmas, such as the difficulty of culturing them and the necessity of producing plant or insect hosts to maintain them for scientific purposes, several phytoplasma molecular and biological properties have been elucidated. Further research work, including the development of effective and ecofriendly strategies to control phytoplasma-associated diseases, will greatly contribute to both the understanding of phytoplasma biology and their physiopathological role in agricultural productions.

## Figures and Tables

**Table 1:** Molecular diversity and geographic distribution of selected phytoplasmas belonging to different ribosomal groups/‘*Candidatus* Phytoplasma’ species (marked by different color) associated with witches’ broom symptoms.

Disease (Acronym)	Continent	16Sr Subgroups	‘*Candidatus* Phytoplasma’ Species	GenBank Accession Number	References
Aster yellows w. b. (AY-WB)	America	16SrI-A	‘*Ca*. P. asteris’	NC_007716	[13]
Paulownia w. b. (PaWB)	Asia	16SrI-D		AY265206	[14]
Strawberry witches’ broom (STRAWB1), (STRAWB2)	America	16SrI-I / -K		U96614, U96616	[15]
Peach rosette-like (PRU0382)	America	16SrI-W		HQ450211	[16]
Peanut witches’ broom (PnWB)	America	16SrII-A		L33765	[17]
Lime witches’ broom (WBDL)	Asia	16SrII-B	‘*Ca*. P. aurantifolia’	U15442	[18]
Cactus witches’ broom (CWB)	Asia	16SrII-G to -L		EU099568, EU099552, EU099569, EU099572, EU099551, EU099546, EF647744	[19]
Tabebuia witches’ broom	America	16SrII-O			[20]
Tomatillo witches’ broom	America	16SrII-T		U125185	[21]
Walnut witches’ broom (WWB)	America	16SrIII-G		AF190226, AF190227	[22]
Poinsettia branch-inducing (PoiBI)	Europe, America	16SrIII-H		AF190223	[22]
Chayote w. b. (ChWBIII)	America	16SrIII-J		AF147706	[23]
Black raspberry w. b. (BRWB7)	America	16SrIII-Q		AF302841	[24]
Conyza witches’ broom	America	16SrIII-X		KC412026	[25]
Jujube witches’ broom (JWB-G1)	Asia	16SrV-B	‘*Ca*. P. ziziphi’	AB052876	[26]
*Balanites triflora* w. b. (BltWB)	Asia	16SrV-F	*Ca*. P. balanitae’	AB689678	[27]
Korean jujube witches’ broom	Asia	16SrV-G		AB052879	[26]
*Bischofia polycarpa* witches’ broom	Asia	16SrV-H		KJ452547	[28]
Blackberry witches’ broom	Europe	16SrV-I		KR233473	[29]
Clover proliferation (CP)	America	16SrVI-A	‘*Ca*. P. trifolii’	AY390261	[30]
Erigeron witches’ broom (ErWB)	America	16SrVII-B		AY034608	[31]
Argentinian alfalfa w.b. (ArAWB)	America	16SrVII-C		AY147038	[32]
Erigeron w. b. (EboWB-Br0)	America	16SrVII-D		KJ831066	[33]
Loofah witches’ broom (LufWB)	Asia	16Sr VIII-A	‘*Ca*. P. luffae’	AF086621	[34]
Pigeon pea w. b. (PPWB)	America	16SrIX-A		AF248957	[35]
Almond witches’ broom (AlWB)	Asia	16SrIX-B/-D	‘*Ca*. P. phoenicium’	AF515636, AF515637	[36]
Juniperus witches’ broom	America	16SrIX-E		GQ925918	[37]
Almond and stone fruit witches’ broom (N27-2), (A1-1)	Asia	16SrIX-F/-G	‘*Ca*. P. phoenicium’	HQ407532, HQ407514	[38]
Apple proliferation (AP)	Europe, Asia	16SrX-A	‘*Ca*. P. mali’	AJ542541	[39]
Spartium witches’ broom (SpaWB)	Europe	16SrX-D	‘*Ca*. P. spartii’	X92869	[40]
Black alder w. b. (BAWB, BWB)	Europe	16SrX-E		X76431	[41]
Hibiscus witches’ broom (HibWB)	America, Asia	16SrXV-A	‘*Ca*. P. brasiliense’	AF147708	[42]
Guazuma w. b. (GWB)	America	16SrXV-B		HQ258882	[43]
Chestnut witches’ broom	Asia	16SrXIX-A	‘*Ca*. P. castaneae’	AB054986	[44]
Rhamnus witches’ broom	Europe	16SrXX-A	‘*Ca*. P. rhamni’	AJ583009	[40]
Weeping tea witches’ broom	Oceania	16SrXXV-A *		AF521672	[45]
Cassia w. b. (CaWB)	Asia	16SrXXIX-A	‘*Ca*. P. omanense’	EF666051	[46]
Bindweed witches’ broom (RBiWB)	Asia	16SrXXIX-B		KY047493	[47]
Salt cedar witches’ broom	Asia	16SrXXX-A	‘*Ca*. P. tamaricis’	FJ432664	[48]

w. b., witches’ broom; *, described as sequence deposited in GenBank only.

**Table 2:** Molecular diversity and geographic distribution of selected phytoplasmas belonging to different ribosomal groups/‘*Candidatus* Phytoplasma’ species (marked by different color) associated with little leaf and stunting symptoms.

Disease (Acronym)	Continent	16Sr Subgroups	‘*Candidatus* Phytoplasma’ Species	GenBank Accession Number	References
Blue dwarf wheat (BDW)	Asia	16SrI-C	‘*Ca*. P. tritici’	DQ078304	[50]
Blueberry stunt (BBS3)	America	16SrI-E		AY265213	[14]
Cherry little leaf (ChLL)	Europe	16SrI-Q		AY034089	[51]
Pepper little leaf (PeLL)	America	16SrI-S		DQ092321	[52]
Tomato little leaf (ToLL)	America	16SrI-T		DQ375238	[52]
*Vasconcellea cundinamarcensis* little leaf	China	16SrII-U		KP057205	[53]
Spiraea stunt (SP1)	America	16SrIII-E		AF190228	[23]
*Heterothalamus* little leaf (HetLL)	America	16SrIII-W		KC412029	[26]
Broccoli stunt (BSP-21)	America	16SrIII-Z		JX626327	[22]
Rubus stunt (RuS)	Europe	16SrV-E	‘*Ca*. P. rubi’	AY197648	[54]
*Fragaria multicipita*, multiplier disease	America	16SrVI-B		AF190224	[15]
Periwinkle little leaf (PLL-Bd)	Asia	16SrVI-D		AF228053	[55]
Portulaca little leaf (PLL-Ind)	Asia	16SrVI-H		EF651786	[56]
Soybean stunt (SoyST1c1)	America	16SrXXXI-A	‘*Ca*. P. costaricanum’	HQ225630	[57]

**Table 3:** Molecular diversity and geographic distribution of selected phytoplasmas belonging to different ribosomal groups/‘*Candidatus* Phytoplasma’ species (marked by different color) and associated with phyllody and virescence symptoms.

Disease (Acronym)	Continent	16Sr Subgroups	‘*Candidatus* Phytoplasma’ Species	GenBank Accession Number	References
Clover phyllody (CPh)	America	16SrI-C		AF222065	[15]
Faba bean phyllody (FBP)	Asia, Africa	16SrII-C		X83432	[58]
*Pichris echioides* phyllody (PEY)	Europe	16SrII-E		Y16393	[58]
Cotton phyllody (CoP)	Africa	16SrII-F		EF186827	[59]
Strawberry leafy fruit (SLF)	America	16SrIII-K		AF274876	[15]
Dandelion virescence (DanVir)	Europe	16SrIII-O/-P		AF370120, AF370119	[60]
*Heterothalamus* little leaf (HetLL)	America	16SrIII-W		KC412029	[26]
*Centarurea solstitialis* virescence (CSVI)	Europe	16SrVI-E		AY270156	[61]
Catharanthus phyllody (CPS)	Africa	16SrVI-F		EF186819	[59]
Naxos periwinkle virescence (NAXOS)	Europe, Asia, America	16SrIX-C		HQ589191	[62]
Sarsoon phyllody	Asia	16SrIX-H		KU892213	[63]
Japanese hydrangea phyllody	Asia	16SrXII-D	‘*Ca*. P. japonicum’	AB010425	[64]
Mexican periwinkle virescence (MPV)	America	16SrXIII-A	‘*Ca*. P. hispanicum’	AF248960	[65]
Strawberry green petal (STRAWB2)	America	16SrXIII-B		U96616	[15]
Malaysian periwinkle virescence (MaPV)	Asia	16SrXXXII-A	‘*Ca*. P. malaysianum’	EU371934	[66]

**Table 4:** Molecular diversity and geographic distribution of selected phytoplasmas belonging to different ribosomal groups/‘*Candidatus* Phytoplasma’ species (marked by different color) associated with yellows and decline symptoms.

Disease (Acronym)	Continent	16Sr Subgroups	‘*Candidatus* Phytoplasma’ Species	GenBank Accession Number	References
Aster yellows (MAY)	America	16SrI-B	‘*Ca.* P. asteris’	M30790	[14]
Aster yellows apricot Spain (A-AY)	Europe, America	16SrI-F		AY265211	[14]
Aster yellows (AV2192	Europe	16SrI-L		AY180957	[67]
Aster yellows (AVUT)	Europe	16SrI-M		AY265209	[17]
Aster yellows (IoWB)	America	16SrI-N		AY265205	[17]
Aster yellows from *Populus* (PopAY)	Europe	16SrI-P		AF503568	[68]
Papaya mosaic (PpM)	Oceania	16SrII-D	‘*Ca*. P. australasia’	Y10096	[69]
*Echinopsis* yellow patch	America	16SrII-R		DQ535900	[21]
Peach X-disease (PX11CT1)	America	16SrIII-A	‘*Ca*. P. pruni’	JQ044393	[22]
Clover yellow edge (CYE)	America, Europe	16SrIII-B		AF173558	[22]
Goldenrod yellows (GR1)	America	16SrIII-D		GU004372	[22]
Milkweed yellows (MW1)	America	16SrIII-F		AF510724	[22]
Virginia grapevine yellows (VGYIII)	America	16SrIII-I		AF060875	[70]
Western peach X-disease (WX)	America	16SrIII-S		L04682	[71]
Coconut lethal yellowing (LYJ-C8)	America	16SrIV-A	‘*Ca*. P. palmae’	AF498307	[4]
Yucatan coconut lethal decline (LDY)	America	16SrIV-B		U18753	[72]
Tanzanian coconut lethal decline (LDT)	Africa	16SrIV-C	‘*Ca*. P. cocostanzaniae’	X80117	[72]
Texas phoenix decline (TPD	America	16SrIV-D		AF434969	[73]
Coconut lethal yellowing (LYDR-B5)	America	16SrIV-E		DQ631639	[74]
*Washingtonia robusta* decline	America	16SrIV-F		EU241512	[73]
Elm yellows (EY)	Europe, America	16SrV-A	‘*Ca*. P. ulmi’	AY197655	[75]
‘Flavescence dorée’ (FD-C)	Europe	16SrV-C		X76560	[76]
‘Flavescence dorée’ (FD-D)	Europe	16SrV-D		AJ548787	[76]
Illinois elm yellows (EY-IL1)	America	16SrVI-C		AF409069	[77]
Ash yellows (AshY)	America, Europe, Asia	16SrVII-A	‘*Ca*. P. fraxini’	AF092209	[78]
European stone fruit yellows (ESFY)	Europe, Asia	16SrX-B	‘*Ca*. P. prunorum’	AJ542544	[39]
Pear decline (PD)	Europe, America	16SrX-C	‘*Ca*. P. pyri’	AJ542543	[39]
Rice yellow dwarf (RYD)	Asia	16SrXI-A	‘*Ca*. P. oryzae’	AB052873	[79]
”Stolbur” (STOL11)	Europe, America, Asia, Africa	16SrXII-A	’*Ca*. P. solani’	AF248959	[80]
Australian grapevine yellows (AUSGY)	Oceania	16SrXII-B	‘*Ca*. P. australiense’	L76865	[81]
Strawberry lethal yellows (StrawLY)	Oceania	16SrXII-C		AJ243045	[82]
Yellows diseased strawberry (StrawY)	Europe	16SrXII-E	‘*Ca*. P. fragariae’	DQ086423	[83]
“Bois noir” (BN-Op30), (BN-Fc3)	Europe	16SrXII-F /-G		EU836652, EU836647	[84]
Bindweed yellows (BY-S57/11)	Europe	16SrXII-H	‘*Ca*. P. convolvuli’	JN833705	[85]
Chinaberry yellows (CBY1)	America	16SrXIII-C		AF495882	[86]
Chinaberry yellowing (ChTY)	America	16SrXIII-G	‘*Ca.* P. meliae’	KU850940	[86]
Sugarcane yellow leaf syndrome	America	16SrXVI-A	‘*Ca*. P. graminis’	AY725228	[87]
Pinus phytoplasma (PinP)	Europe, America, Africa	16SrXXI-A	‘*Ca.* P. pini’	AJ310849	[88]
Lethal yellowing Mozambique (LYDM 178)	Africa	16SrXXII-A	‘*Ca*. P. palmicola’	KF751387	[89]
Cape Saint Paul Wilt Ghana (LDG)	Africa	16SrXXII-B		Y13912	[90]
Buckland valley grapevine yellows	Oceania	16SrXXIII-A *		AY083605	[45]
Malayan yellow dwarf (MYD)	Asia	16SrXXXII-B		EU498727	[66]
Malayan oil palm (MOP)	Asia	16SrXXXII-C		EU498728	[66]
Allocasuarina phytoplasma	Oceania	16SrXXXIII-A	‘*Ca.* P. allocasuarinae’	AY135523	[40]
Bogia coconut syndrome (BCS)	Oceania	Not determ.	‘*Ca*. P. noviguineense’	LC228755	[91]
Palm decline (RID7692)	Oceania	Not determ.	‘*Ca*. P. dypsidis’	MT233886	[92]
Not described	Oceania	Not determ.	‘*Ca*. P. stylosanthis’	MT431550	[93]
Palm decline	Oceania	16SrXXXVI	‘*Ca*. P. wodyetiae’	KY069029	[94]

*, described as sequence deposited in GenBank only.

**Table 5:** Molecular diversity and geographic distribution of selected phytoplasmas belonging to different ribosomal groups/‘*Candidatus* Phytoplasma’ species (marked by different color) associated with white leaf symptoms.

Disease (Acronym)	Continent	16Sr Subgroups	‘*Candidatus* Phytoplasma’ Species	GenBank Accession Number	References
Cirsium white leaf (CirWL)	Europe	16SrIII-R		AF373105	[72]
Cirsium white leaf (CWL)	Europe	16SrIII-U		AF373105, AF373106	[81]
Sugarcane white leaf (SCWL)	Asia	16SrXI-B	‘*Ca*. P. sacchari’	X76432	[96]
Sugarcane white leaf (SCWL)	Asia	16SrXI-D		KR020685	[97]
Bermudagrass white leaf (BGWL)	Europe	16SrXIV-A	‘*Ca*. P. cynodontis’	AJ550984	[98]
Bermudagrass white leaf Iran	Asia	16SrXIV-B		EF444485	[99]
Bermudagrass white leaf (RS304/13)	Europe	16SrXIV-C		KP019339	[100]

**Table 6:** Molecular diversity and geographic distribution of selected phytoplasmas belonging to different ribosomal groups/‘*Candidatus* Phytoplasma’ species (marked by different color) associated with purple top and other malformations.

Disease (Acronym)	Continent	16Sr Subgroups	‘*Candidatus* Phytoplasma’ Species	GenBank Accession Number	References
Soybean purple stem (SPS)	America	16SrI-O		AF268405	[101]
Mexican potato purple top (JAL8), (SON18)	America	16SrI-U/-V		FJ914650, FJ914642	[53]
Papaya bunchy top (BTS)	America	16SrI-X		JF781308	[102]
Tomato “brote grande”	America	16SrI-Y	‘*Ca.* P. lycopersici’	EF199549	[103]
Papaya bunchy top (BTS)	America	16SrI-Z		JF781311	[104]
Potato purple top	Asia	16SrII-M		FJ914643	[105]
Papaya BTSp	America	16SrII-N		JF781309	[102]
Cuban papaya	America	16SrII-P		DQ286948	[22]
Papaya bunchy top (TSpHav02-IIA)	America	16SrII-Q		JF78131	[22]
*Echinopsis* yellow patch	America	16SrII-R		DQ535900	[22]
*Amaranthus hypochondriacus* 52A	America	16SrII-S		FJ357164	[22]
Pecan bunch (PB)	America	16SrIII-C		GU004371	[23]
Cassava frog skin (CFSD)	America	16SrIII-L		EU346761	[106]
Potato purple top (MT117)	America	16SrIII-M		FJ226074	[23]
Potato purple top (AKpot6)	America	16SrIII-N		GU004365	[23]
Sweet and sour cherry (ChD)	Europe	16SrIII-T		FJ231728	[107]
Passion fruit phytoplasma (PassWB-Br4)	America	16SrIII-V		GU292082	[108]
Cranberry false-blossom	America	16SrIII-Y		KF62652	[109]
Passionfruit (WB-Br4)	America	16SrVI-I	‘*Ca*. P. sudamericanum’	GU292081	[110]
Leafhopper-borne (BVK)	Europe	16SrXI-C		X76429	[13]
Cirsium phytoplasma	Europe	16SrXI-E	‘*Ca*. P. cirsii’	KR869146	[111]
Sugarcane grassy shoot (SCGS)	Asia	16SrXI-F		HF586636	[112]
Potato (169/Hezuo 88)	Asia	16SrXII-I		EU338445	[112]
Mexican potato purple top (SINPV)	America	16SrXIII-D		FJ914647	[53]
Papaya apical curl necrosis (PACN)	America	16SrXIII-E		EU719111	[113]
Strawberry red leaf	America	16SrXIII-F		KJ921641	[114]
Papaya bunchy top	America	16SrXVII-A	‘*Ca*. P. caricae’	AY725234	[88]
American potato purple top wilt	America	16SrXVIII-A	‘*Ca*. P. americanum’	DQ174122	[115]
Sorghum bunchy shoot	Oceania	16SrXXIV-A *		AF509322	[45]
Sugarcane phytoplasma D3T1	Africa	16SrXXVI-A *		AJ539179	[45]
Sugarcane phytoplasma D3T2	Africa	16SrXXVII-A *		AY539180	[45]
Derbid phytoplasma	Africa	16SrXXVIII-A *		AY744945	[45]

*, described as sequence deposited in GenBank only.

## References

[1] McCann H.C., Li L., Liu Y., Li D., Pan H., Zhong C., Rikkerink E., Templeton M.D., Straub C., Colombi E. (2017). Origin and evolution of the kiwifruit canker pandemic. Gen. Biol. Evol..

[2] Bové J.-M. (2006). Huanglongbing: A destructive, newly-emerging, century-old disease of citrus. J. Plant Pathol..

[3] IRPCM (2004). ‘*Candidatus* Phytoplasma’, a taxon for the wall-less, non-helical prokaryotes that colonise plant phloem and insects. Int. J. Syst. Evol. Microbiol..

[4] Bertaccini A., Arocha-Rosete Y., Contaldo N., Duduk B., Fiore N., Montano H.G., Kube M., Kuo C.-H., Martini M., Oshima K. (2022). Revision of the ‘*Candidatus* Phytoplasma’ species description guidelines. Int. J. Syst. Evol. Microbiol..

[5] Namba S. (2019). Molecular and biological properties of phytoplasmas. Proc. Jpn. Acad. Ser. B Phys. Biol. Sci..

[6] Doi Y., Teranaka M., Yora K., Asuyama H. (1967). Mycoplasma or PLT grouplike microrganisms found in the phloem elements of plants infected with mulberry dwarf, potato witches’ broom, aster yellows or pawlownia witches’ broom. Ann. Phytopath. Soc. Jpn..

[7] Contaldo N., Bertaccini A., Paltrinieri S., Windsor H.M., Windsor G.D. (2012). Axenic culture of plant pathogenic phytoplasmas. Phytopath. Medit..

[8] Contaldo N., Satta E., Zambon Y., Paltrinieri S., Bertaccini A. (2016). Development and evaluation of different complex media for phytoplasma isolation and growth. J. Microbiol. Meth..

[9] Contaldo N., D’Amico G., Paltrinieri S., Diallo H.A., Bertaccini A., Arocha Rosete Y. (2019). Molecular and biological characterization of phytoplasmas from coconut palms affected by the lethal yellowing disease in Africa. Microbiol. Res..

[10] Luis Pantoja M., Paredes-Tomás C., Uneau Y., Myrie W., Morillon R., Satta E., Contaldo N., Pacini F., Bertaccini A. (2021). Identification of ‘*Candidatus* Phytoplasma’ species in “huanglongbing” infected citrus orchards in the Caribbean. Eur. J. Plant Pathol..

[11] Betancourt C., Pardo J., Muñoz J., Alvarez E. (2019). Isolation of phytoplasmas associated to frogskin disease in cassava. Rev. UDCA Actual. Divulg. Cient..

[12] Lee I.-M., Gundersen-Rindal D.E., Davis R.E., Bartoszyk I.M. (1998). Revised classification scheme of phytoplasmas based on RFLP analyses of 16S rRNA and ribosomal protein gene sequences. Int. J. Syst. Evol. Microbiol..

[13] Bai X., Zhang J., Ewing A., Miller S.A., Radek A.J., Shevchenko D.V., Tsukerman K., Walunas T., Lapidus A., Campbell J.W. (2006). Living with genome instability: The adaptation of phytoplasmas to diverse environments of their insect and plant hosts. J. Bacteriol..

[14] Lee I.-M., Gundersen-Rindal D.E., Davis R.E., Bottner K.D., Marcone C., Seeműller E. (2004). ‘*Candidatus* Phytoplasma asteris’, a novel taxon associated with aster yellows and related diseases. Int. J. Syst. Evol. Microbiol..

[15] Jomantiene R., Davis R.E., Maas J., Dally E.L. (1998). Classification of new phytoplasmas associated with diseases of strawberry in Florida, based on analysis of 16S rRNA and ribosomal protein gene operon sequences. Int. J. Syst. Evol. Microbiol..

[16] Arocha-Rosete Y., Zunnoon-Khan S., Krukovets I., Crosby W., Scott J., Bertaccini A., Michelutti R. (2011). Identification and molecular characterization of the phytoplasma associated with peach rosette-like disease at the Canadian clonal Genebank based on the 16S rRNA gene analysis. Can. J. Plant Pathol..

[17] Gundersen D.E., Lee I.-M., Rehner S.A., Davis R.E., Kingsbury D.T. (1994). Phylogeny of mycoplasmalike organisms (phytoplasmas): A basis for their classification. J. Bacteriol..

[18] Zreik L., Carle P., Bové J.-M., Garnier M. (1995). Characterization of the mycoplasmalike organism associated with witches’ broom disease of lime and proposition of a ‘*Candidatus*’ taxon for the organism, ‘*Candidatus* Phytoplasma aurantifolia’. Int. J. Syst. Bacteriol..

[19] Cai H., Wei W., Davis R.E., Chen H., Zhao Y. (2008). Genetic diversity among phytoplasmas infecting *Opuntia* species: Virtual RFLP analysis identifies new subgroups in the peanut witches’ broom phytoplasma group. Int. J. Syst. Evol. Microbiol..

[20] Mafia R.G., Barreto R.W., Vanetti C.A., Hodgetts J., Dickinson M., Alfenas A.C. (2007). A phytoplasma is associated with witches’ broom disease of *Tabebuia pentaphylla* in Brazil. New Dis. Rep..

[21] Perez-López E., Luna-Rodríguez M., Olivier C.Y., Dumonceaux T.J. (2016). The underestimated diversity of phytoplasmas in Latin America. Int. J. Syst. Evol. Microbiol..

[22] Davis R.E., Zhao Y., Dally E.L., Lee I.-M., Jomantiene R., Douglas S. (2013). ‘*Candidatus* Phytoplasma pruni’, a novel taxon associated with X-disease of stone fruits, *Prunus* spp.: Multilocus characterization based on 16S rRNA, secY, and ribosomal protein genes. Int. J. Syst. Evol. Microbiol..

[23] Montano H.G., Davis R.E., Dally E.L., Pimentel J.P., Brioso P.S.T. (2000). Identification and phylogenetic analysis of a new phytoplasma from diseased chayote in Brazil. Plant Dis..

[24] Davis R.E., Dally E.L., Converse R.H. (2001). Molecular identification of a phytoplasma associated with witches’-broom disease of black raspberry in Oregon and its classification in group 16SrIII, new subgroup Q. Plant Dis..

[25] Galdeano E., Guzmán F.A., Fernández F., Conci R.G. (2013). Genetic diversity of 16SrIII group phytoplasmas in Argentina. Predominance of subgroups 16SrIII-J and B and two new subgroups 16SrIII-W and X. Eur. J. Plant Pathol..

[26] Jung H.-Y., Sawayanagi T., Kakizawa S., Nishigawa H., Wei W., Oshima K., Miyata S., Ugaki M., Hibi T., Namba S. (2003). ‘*Candidatus* Phytoplasma ziziphi’, a novel phytoplasma taxon associated with jujube witches’ broom disease. Int. J. Syst. Evol. Microbiol..

[27] Win N.K.K., Lee S.-Y., Bertaccini A., Namba S., Jung H.-Y. (2013). ‘*Candidatus* Phytoplasma balanitae’ associated with witches’ broom disease of *Balanites triflora*. Int. J. Syst. Evol. Microbiol..

[28] Lai F., Song C.S., Ren Z.G., Lin C.L., Xu Q.C., Li Y., Piao C.G., Yu S.S., Guo M.W., Tian G.Z. (2014). Molecular characterization of a new member of the 16SrV group of phytoplasma associated with *Bischofia polycarpa* (Levl.) Airy Shaw witches’ broom disease in China by a multiple gene-based analysis. Austral. Plant Pathol..

[29] Fránová J., de Sousa E., Mimoso C., Cardoso F., Contaldo N., Paltrinieri S., Bertaccini A. (2016). Multigene characterization of a new ‘*Candidatus* Phytoplasma rubi’-related strain associated with blackberry witches’ broom in Portugal. Int. J. Syst. Evol. Microbiol..

[30] Hiruki C., Wang K. (2004). Clover proliferation phytoplasma: ‘*Candidatus* Phytoplasma trifolii’. Int. J. Syst. Evol. Microbiol..

[31] Barros T.S.L., Davis R.E., Resende R.O., Dally E.L. (2002). Erigeron witches’ broom phytoplasma in Brazil represents new subgroup VII-B in 16S rRNA gene group VII, the ash yellows phytoplasma group. Plant Dis..

[32] Conci L., Meneguzzi N., Galdeano E., Torres L., Nome C., Nome S. (2005). Detection and molecular characterisation of an alfalfa phytoplasma in Argentina that represents a new subgroup in the 16S rDNA ash yellows group (‘*Candidatus* Phytoplasma fraxini’). Eur. J. Plant Pathol..

[33] Flôres D., Amaral Mello A.O., Pereira T.B.C., Rezende J.A.M., Bedendo I.P. (2015). A novel subgroup 16SrVII-D phytoplasma identified in association with erigeron witches’ broom. Int. J. Syst. Evol. Microbiol..

[34] Davis R.E., Zhao Y., Wei W., Dally E.L., Lee I.-M. (2017). ‘*Candidatus* Phytoplasma luffae’, a novel taxon associated with witches’ broom disease of loofah, *Luffa aegyptica* Mill. Int. J. Syst. Evol. Microbiol..

[35] Gundersen D.E., Lee I.-M., Schaff D.A., Harrison N.A., Chang C.J., Davis R.E., Kinsbury D.T. (1996). Genomic diversity among phytoplasma strains in 16S rRNA group I (aster yellows and related phytoplasmas) and III (X-disease and related phytoplasmas). Int. J. Syst. Bacteriol..

[36] Verdin E., Salar P., Danet J.-L., Choueiri E., Jreijiri F., El Zammar S., Gélie B., Bové J.-M., Garnier M. (2003). ‘*Candidatus* Phytoplasma phoenicium’ sp. nov., a novel phytoplasma associated with an emerging lethal disease of almond trees in Lebanon and Iran. Int. J. Syst. Evol. Microbiol..

[37] Davis R.E., Dally E., Zhao Y., Lee I.-M., Jomantiene R., Detweiler A.J., Putnam M.L. (2010). First report of a new subgroup 16SrIX-E (‘*Candidatus* Phytoplasma phoenicium’-related) phytoplasma associated with juniper witches’ broom disease in Oregon, USA. Plant Pathol..

[38] Molino Lova M., Quaglino F., Abou-Jawdah Y., Choueiri E., Sobh H., Casati P., Tedeschi R., Alma A., Bianco P.A. (2011). Identification of new 16SrIX subgroups, -F and -G, among ‘*Candidatus* Phytoplasma phoenicium’ strains infecting almond, peach and nectarine in Lebanon. Phytopath. Medit..

[39] Seemüller E., Schneider B. (2004). ‘*Candidatus* Phytoplasma mali’, ‘*Candidatus* Phytoplasma pyri’ and ‘*Candidatus* Phytoplasma prunorum’, the causal agents of apple proliferation, pear decline and European stone fruit yellows, respectively. Int. J. Syst. Evol. Microbiol..

[40] Marcone C., Gibb K.S., Streten C., Schneider B. (2004). ‘*Candidatus* Phytoplasma spartii’, ‘*Candidatus* Phytoplasma rhamni’ and ‘*Candidatus* Phytoplasma allocasuarinae’, respectively associated with *Spartium* witches’ broom, buckthorn witches’ broom and *Allocasuarina* yellows diseases. Int. J. Syst. Evol. Microbiol..

[41] Seemüller E., Schneider B., Maurer R., Ahrens U., Daire X., Kison H., Lorenz K., Firrao G., Avinent L., Sears B.B. (1994). Phylogenetic classification of phytopathogenic mollicutes by sequence analysis of 16S ribosomal DNA. Int. J. Syst. Bacteriol..

[42] Montano H.G., Davis R.E., Dally E.L., Hogenhout S., Pimentel J.P., Brioso P.S. (2001). ‘*Candidatus* Phytoplasma brasiliense’, a new phytoplasma taxon associated with hibiscus witches’ broom disease. Int. J. Syst. Evol. Microbiol..

[43] Villalobos W., Martini M., Garita L., Muñoz M., Osler R., Moreira L. (2011). *Guazuma ulmifolia* (Sterculiaceae), a new natural host of 16SrXV phytoplasma in Costa Rica. Trop. Plant Pathol..

[44] Jung H.-Y., Sawayanagi T., Kakizawa S., Nishigawa H., Miyata S., Oshima K., Ugaki M., Lee J.T., Hibi T., Namba S. (2002). ‘*Candidatus* Phytoplasma castaneae’, a novel phytoplasma taxon associated with chestnut witches’ broom disease. Int. J. Syst. Evol. Microbiol..

[45] Wei W., Davis R.E., Lee I.-M., Zhao Y. (2007). Computer-simulated RFLP analysis of 16S rRNA genes: Identification of ten new phytoplasma groups. Int. J. Syst. Evol. Microbiol..

[46] Al-Saady N.A., Khan A.J., Calari A., Al-Subhi A.M., Bertaccini A. (2008). ‘*Candidatus* Phytoplasma omanense’, associated with witches’ broom of *Cassia italica* (Mill.) Spreng in Oman. Int. J. Syst. Evol. Microbiol..

[47] Esmailzadeh Hosseini S.A., Salehi M., Mirchenari S.M., Contaldo N., Paltrinieri S., Bertaccini A. (2016). Occurrence of a ‘*Candidatus* Phytoplasma omanense’-related strain in bindweed showing a witches’ broom disease in Iran. Phytopath. Moll..

[48] Zhao Y., Sun Q., Wei W., Davis R.E., Wu W., Liu Q. (2009). ‘*Candidatus* Phytoplasma tamaricis’, a novel taxon discovered in witches’-broom-diseased salt cedar (*Tamarix chinensis* Lour.). Int. J. Syst. Evol. Microbiol..

[49] Duduk B., Tian J.B., Contaldo N., Fan X.P., Paltrinieri S., Chen Q.F., Zhao Q.F., Bertaccini A. (2010). Occurrence of phytoplasmas related to “stolbur” and to ‘*Candidatus* Phytoplasma japonicum’ in woody host plants in China. J. Phytopath..

[50] Zhao Y., Wei W., Davis R.E., Lee I.-M., Bottner-Parker K.D. (2021). The agent associated with blue dwarf disease in wheat represents a new phytoplasma taxon, ‘*Candidatus* Phytoplasma tritici’. Int. J. Syst. Evol. Microbiol..

[51] Valiunas D., Jomantiene R., Davis R.E. (2005). A ‘*Candidatus* Phytoplasma asteris’-related phytoplasma associated with cherry little leaf disease represents a new subgroup, 16SrI-Q. Phytopathology.

[52] Santos-Cervantes M.E., Chávez-Medina J.A., Acosta-Pardini J., Flores-Zamora G.L., Méndez-Lozano J., Leyva-López N.E. (2010). Genetic diversity and geographical distribution of phytoplasmas associated with potato purple top disease in Mexico. Plant Dis..

[53] Yang Y., Jiang L., Che H., Cao X., Luo D. (2016). Identification of a novel subgroup 16SrII-U phytoplasma associated with papaya little leaf disease. Int. J. Syst. Evol. Microbiol..

[54] Malembic-Maher S., Salar P., Filippin L., Carle P., Angelini E., Foissac X. (2011). Genetic diversity of European phytoplasmas of the 16SrV taxonomic group and proposal of ‘*Candidatus* Phytoplasma rubi’. Int. J. Syst. Evol. Microbiol..

[55] Siddique A.B.M., Agrawal G.K., Alam N., Krishina Reddy M. (2001). Electron microscopy and molecular characterization of phytoplasmas associated with little leaf disease of brinjal (*Solanum melongena*) and periwinkle (*Catharanthus roseus*) in Bangladesh. J. Phytopath..

[56] Samad A., Ajayakumar P.V., Shasany A.K., Gupta M.K., Alam M., Rastogi S. (2008). Occurrence of a clover proliferation (16SrVI) group phytoplasma associated with little leaf disease of *Portulaca grandiflora* in India. Plant Dis..

[57] Lee I.-M., Bottner-Parker K.D., Zhao Y., Villalobos W., Moreira L. (2011). ‘*Candidatus* Phytoplasma costaricanum’ a novel phytoplasma associated with an emerging disease in soybean (*Glycine max*). Int. J. Syst. Evol. Microbiol..

[58] Seemüller E., Marcone C., Lauer U., Ragozzino A., Göschl M. (1998). Current status of molecular classification of the phytoplasmas. J. Plant Pathol..

[59] Martini M., Lee I.-M., Bottner K.D., Zhao Y., Botti S., Bertaccini A., Harrison N.A., Carraro L., Marcone C., Khan J. (2007). Ribosomal protein gene-based phylogeny for finer differentiation and classification of phytoplasmas. Int. J. Syst. Evol. Microbiol..

[60] Jomantiene R., Maas J.L., Takeda F., Davis R.E. (2002). Molecular identification and classification of strawberry phylloid fruit phytoplasma in group 16SrI, new subgroup. Plant Dis..

[61] Faggioli F., Pasquini G., Lumia V., Campobasso G., Widmer T.L., Quimby P.C. (2004). Molecular identification of a new member of the clover proliferation phytoplasma group (16SrVI) associated with yellow starthistle virescence in Italy. Eur. J. Plant Pathol..

[62] Duduk B., Mejia J.F., Calari A., Bertaccini A. Identification of 16SrIX group phytoplasmas infecting Colombian periwinkles and molecular characterization on several genes. Proceedings of the 17th IOM Congress.

[63] Ahmad J.N., Ahmad S.J.N., Irfan M., Paltrinieri S., Contaldo N., Bertaccini A. (2017). Molecular detection, identification, characterization and transmission study of sarsoon phyllody in Punjab–Pakistan associated with phytoplasmas affiliated to the new subgroup 16SrIX-H. Eur. J. Plant Pathol..

[64] Sawayanagi T., Horikoshi N., Kanehira T., Shinohara M., Bertaccini A., Cousin M.-T., Hiruki C., Namba S. (1999). ‘*Candidatus* Phytoplasma japonicum’, a new phytoplasma taxon associated with Japanese hydrangea phyllody. Int. J. Syst. Evol. Microbiol..

[65] Davis R.E., Harrison N.A., Zhao Y., Wei W., Dally E.L. (2016). ‘*Candidatus* Phytoplasma hispanicum’, a novel taxon associated with Mexican periwinkle virescence disease of *Catharanthus roseus*. Int. J. Syst. Evol. Microbiol..

[66] Nejat N., Vadamalai G., Davis R.E., Harrison N.A., Sijam K., Dickinson M., Abdullah S.N.A., Zhao Y. (2013). ‘*Candidatus* Phytoplasma malaysianum’, a novel taxon associated with virescence and phyllody of Madagascar periwinkle (*Catharanthus roseus*). Int. J. Syst. Evol. Microbiol..

[67] Lee I.-M., Martini M., Bottner K.D., Dane R.A., Black M.C., Troxclair N. (2003). Ecological implications from a molecular analysis of phytoplasmas involved in an aster yellows epidemic in various crops in Texas. Phytopathology.

[68] Šeruga M., Škorić D., Botti S., Paltrinieri S., Juretić N., Bertaccini A. (2003). Molecular characterization of a phytoplasma from the aster yellows (16SrI) group naturally infecting *Populus nigra* L. ‘Italica’ trees in Croatia. For. Pathol..

[69] White D.T., Blackall L.L., Scott P.T., Walsh K.B. (1998). Phylogenetic positions of phytoplasmas associated with dieback, yellow crinkle and mosaic diseases of papaya, and their proposed inclusion in ‘*Candidatus* Phytoplasma australiense’ and a new taxon, ‘*Candidatus* Phytoplasma australasia’. Int. J. Syst. Bacteriol..

[70] Davis R.E., Jomantiene R., Dally E.L., Wolf T.K. (1998). Phytoplasmas associated with grapevine yellows in Virginia belong to group 16SrI, subgroup A (tomato big bud phytoplasma subgroup), and group 16SrIII, new subgroup I. Vitis.

[71] Zhao Y., Wei W., Lee I.-M., Shao J., Suo X., Davis R.E. (2009). Construction of an interactive online phytoplasma classification tool, iPhyClassifier, and its application in analysis of the peach X-disease phytoplasma group (16SrIII). Int. J. Syst. Evol. Microbiol..

[72] Harrison N.A., Richardson P.A., Kramer J.B., Tsai J.H. (1994). Detection of the mycoplasma-like organism associated with lethal yellowing disease of palms in Florida by polymerase chain reaction. Plant Pathol..

[73] Harrison N.A., Helmick E.E., Elliott M.L. (2008). Lethal yellowing-type diseases of palms associated with phytoplasmas newly identified in Florida, USA. Ann. Appl. Biol..

[74] Martinez R.T., Narvaez M., Fabre S., Harrison N.A., Oropeza C., Dollet M., Hichez E. (2007). Coconut lethal yellowing on the southern coast of the Dominican Republic is associated with a new 16SrIV group phytoplasma. New Dis. Rep..

[75] Lee I.-M., Martini M., Marcone C., Zhu S.F. (2004). Classification of phytoplasma strains in the elm yellows group (16SrV) and proposal of ‘*Candidatus* Phytoplasma ulmi’ for the phytoplasma associated with elm yellows. Int. J. Syst. Evol. Microbiol..

[76] Martini M., Murari E., Mori N., Bertaccini A. (1999). Identification and epidemic distribution of two “flavescence dorée”-related phytoplasmas in Veneto (Italy). Plant Dis..

[77] Jacobs K.A., Lee I.-M., Griffiths H.M., Miller F.D., Bottner K.D. (2003). A new member of the clover proliferation phytoplasma group (16SrVI) associated with elm yellows in Illinois. Plant Dis..

[78] Griffiths H.M., Sinclair W.A., Smart C.D., Davis R.E. (1999). The phytoplasma associated with ash yellows and lilac witches’ broom: ‘*Candidatus* Phytoplasma fraxini’. Int. J. Syst. Bacteriol..

[79] Jung H.-Y., Sawayanagi T., Wongkaew P., Kakizawa S., Nishigawa H., Wei W., Oshima K., Miyata S., Ugaki M., Hibi T. (2003). ‘*Candidatus* Phytoplasma oryzae’, a novel phytoplasma taxon associated with rice yellow dwarf disease. Int. J. Syst. Evol. Microbiol..

[80] Quaglino F., Zhao Y., Casati P., Bulgari D., Bianco P.A., Wei W., Davis R.E. (2013). ‘*Candidatus* Phytoplasma solani’, a novel taxon associated with “stolbur” and “bois noir”-related diseases of plants. Int. J. Syst. Evol. Microbiol..

[81] Davis R.E., Dally E.L., Gundersen D.E., Lee I.-M., Habili N. (1997). ‘*Candidatus* Phytoplasma australiense’ a new phytoplasma taxonb associated with Australian grapevine yellows. Int. J. Syst. Bacteriol..

[82] Padovan A., Gibb K., Persley D. (2000). Association of ’*Candidatus* Phytoplasma australiense’ 38 with green petal and lethal yellows diseases in strawberry. Plant Pathol..

[83] Valiunas D., Staniulis J., Davis R.E. (2006). ‘*Candidatus* Phytoplasma fragariae’, a novel phytoplasma taxon discovered in yellows diseased strawberry, *Fragaria* × *ananassa*. Int. J. Syst. Evol. Microbiol..

[84] Quaglino F., Zhao Y., Bianco P.A., Wei W., Casati P., Durante G., Davis R.E. (2009). New 16Sr subgroups and distinct single nucleotide polymorphism lineages among grapevine “bois noir” phytoplasma populations. Ann. Appl. Biol..

[85] Martini M., Marcone C., Mitrovic J., Maixner M., Delic D., Myrta A., Ermacora P., Bertaccini A., Duduk B. (2012). ‘*Candidatus* Phytoplasma convolvuli’, a new phytoplasma taxon associated with bindweed yellows in four European countries. Int. J. Syst. Evol. Microbiol..

[86] Fernández F.D., Galdeano E., Kornowski M.V., Arneodo J.D., Conci L.R. (2016). Description of ‘*Candidatus* Phytoplasma meliae’, a phytoplasma associated with Chinaberry (*Melia azedarach* L.) yellowing in South America. Int. J. Syst. Evol. Microbiol..

[87] Arocha Y., López M., Piñol B., Fernández M., Picornell B., Almeida R., Palenzuela I., Wilson M.R., Jones P. (2005). ‘*Candidatus* Phytoplasma graminis’ and ‘*Candidatus* Phytoplasma caricae’, two novel phytoplasmas associated with diseases of sugarcane, weeds and papaya in Cuba. Int. J. Syst. Evol. Microbiol..

[88] Schneider B., Torres E., Martín M.P., Schröder M., Behnke H.D., Seemüller E. (2005). ’*Candidatus* Phytoplasma pini’, a novel taxon from *Pinus silvestris* and *Pinus halepensis*. Int. J. Syst. Evol. Microbiol..

[89] Harrison N.A., Davis R.E., Oropeza C., Helmick E.E., Narváez M., Eden-Green S., Dollet M., Dickinson M. (2014). ‘*Candidatus* Phytoplasma palmicola’, associated with a lethal yellowing-type disease of coconut (*Cocos nucifera* L.) in Mozambique. Int. J. Syst. Evol. Microbiol..

[90] Tymon A.M., Jones P., Harrison N.A. (1998). Phylogenetic relationships of coconut phytoplasmas and the development of specific oligonucleotide PCR primers. Ann. Appl. Biol..

[91] Miyazaki A., Shigaki T., Koinuma H., Iwabuchi N., Rauka G.B., Kembu A., Saul J., Watanabe K., Nijo T., Maejima K. (2018). ‘*Candidatus* Phytoplasma noviguineense’, a novel taxon associated with Bogia coconut syndrome and banana wilt disease on the island of New Guinea. Int. J. Syst. Evol. Microbiol..

[92] Jones L.M., Pease B., Perkins S.L., Constable F.E., Kinoti W.M., Warmington D., Allgood B., Powell S., Taylor P., Pearce C. (2021). ‘*Candidatus* Phytoplasma dypsidis’, a novel taxon associated with a lethal wilt disease of palms in Australia. Int. J. Syst. Evol. Microbiol..

[93] Jardim B.R., Kinoti W.M., Tran-Nguyen L.T.T., Gambley C., Rodoni B., Constable F.E. (2021). ‘*Candidatus* Phytoplasma stylosanthis’, a novel taxon with a diverse host range in Australia, characterised using multilocus sequence analysis of 16S rRNA, secA, tuf, and rp genes. Int. J. Syst. Evol. Microbiol..

[94] Naderali N., Nejat N., Vadamalai G., Davis R.E., Wei W., Harrison N.A., Kong L.L., Kadir J., Tan H.-Y., Zhao Y. (2017). ‘*Candidatus* Phytoplasma wodyetiae’, a new taxon associated with yellow decline disease of foxtail palm (*Wodyetia bifurcata*) in Malaysia. Int. J. Syst. Evol. Microbiol..

[95] Tedeschi R., Bertaccini A., Bertaccini A., Weintraub P.G., Rao G.P., Mori N. (2019). Transovarial transmission in insect vectors. Phytoplasmas: Plant Pathogenic Bacteria-II Transmission and Management of Phytoplasma Associated Diseases.

[96] Kirdat K., Tiwarekar B., Thorat V., Sathe S., Shouche Y., Yadav A. (2020). ‘*Candidatus* Phytoplasma sacchari’, a novel taxon-associated with sugarcane grassy shoot (SCGS) disease. Int. J. Syst. Evol. Microbiol..

[97] Zhang R.Y., Li W.F., Huang Y.K., Wang X.Y., Shan H.L., Luo Z.-M., Yin J. (2016). Group 16SrXI phytoplasma strains, including subgroup 16SrXI-B and a new subgroup, 16SrXI-D, are associated with sugar cane white leaf. Int. J. Syst. Evol. Microbiol..

[98] Marcone C., Schneider B., Seemüller E. (2004). ‘*Candidatus* Phytoplasma cynodontis’, the phytoplasma associated with Bermuda grass white leaf disease. Int. J. Syst. Evol. Microbiol..

[99] Salehi M., Izadpanah K., Siampour M., Taghizadeh M. (2009). Molecular characterization and transmission of bermuda grass white leaf phytoplasma in Iran. J. Plant Pathol..

[100] Mitrovic J., Smiljković M., Seemüller E., Reinhardt R., Hüttel B., Büttner C., Bertaccini A., Kube M., Duduk B. (2015). Differentiation of ‘*Candidatus* Phytoplasma cynodontis’ based on 16S rRNA and groEL genes and identification of a new subgroup, 16SrXIV-C. Plant Dis..

[101] Lee M.E., Grau C.R., Lukaesko L.A., Lee I.-M. (2002). Identification of aster yellows phytoplasmas in soybean in Wisconsin based on RFLP analysis of PCR-amplified products (16S rDNAs). Can. J. Plant Pathol..

[102] Acosta K.I., Zamora L., Piñol B.E., Fernández A., Chávez A., Flores G., Méndez J., Santos M., Leyva N., Arocha Y. (2013). Identification and molecular characterization of phytoplasmas and rickettsia pathogens associated with bunchy top symptom (BTS) and papaya bunchy top (PBT) of papaya in Cuba. Crop Prot..

[103] Arocha Y., Antesana O., Montellano E., Franco P., Plata G., Jones P. (2007). ‘*Candidatus* Phytoplasma lycopersici’, a phytoplasma associated with “hoja de perejil” disease in Bolivia. Int. J. Syst. Evol. Microbiol..

[104] Acosta-Pérez K.I., Piñol-Pérez B.E., Zamora-Gutierrez L., Quiñones-Pantoja M.L., Miranda-Cabrera I., Leyva-López N.E., Arocha-Rosete Y. (2017). A phytoplasma representative of a new subgroup 16SrI-Z associated with bunchy top symptoms (BTS) on papaya in Cuba. Rev. Protec. Veg..

[105] Yadav A., Bhale U., Thorat V., Shouche Y. (2014). First report of a new subgroup 16SrII-M ‘*Candidatus* Phytoplasma aurantifolia’ associated with witches’ broom disease of *Tephrosia purpurea* in India. Plant Dis..

[106] Alvarez E., Mejía J.F., Llano G.A., Loke J.B., Calari A., Duduk B., Bertaccini A. (2009). Detection and molecular characterization of a phytoplasma associated with frogskin disease in cassava. Plant Dis..

[107] Valiunas D., Jomantiene R., Ivanauskas A., Abraitis R., Staniene G., Zhao Y., Davis R.E. (2009). First report of a new phytoplasma subgroup, 16SrIII-T, associated with decline disease affecting sweet and sour cherry trees in Lithuania. Plant Dis..

[108] Davis R.E., Zhao Y., Dally E.L., Jomantiene R., Lee I.-M., Wei W., Kitajima E.W. (2012). ‘*Candidatus* Phytoplasma sudamericanum’, a novel taxon, and strain PassWB-Br4, a new subgroup 16SrIII-V phytoplasma, from diseased passion fruit (*Passiflora edulis* f. *flavicarpa* Deg.). Int. J. Syst. Evol. Microbiol..

[109] Lee I.-M., Polashock J., Bottner-Parker K.D., Bagadia P.G., Rodriguez-Saona C., Zhao Y., Davis R.E. (2014). New subgroup 16SrIII-Y phytoplasmas associated with false-blossom diseased cranberry (*Vaccinium macrocarpon*) plants and with known and potential insect vectors in New Jersey. Eur. J. Plant Pathol..

[110] Šafárová D., Zemánek T., Válová P., Navrátil M. (2016). ‘*Candidatus* Phytoplasma cirsii’, a novel taxon from creeping thistle [*Cirsium arvense* (L.) Scop]. Int. J. Syst. Evol. Microbiol..

[111] Yadav A., Thorat V., Deokule S., Shouche Y., Prasad D.T. (2017). New subgroup 16SrXI-F phytoplasma strain associated with sugarcane grassy shoot (SCGS) disease in India. Int. J. Syst. Evol. Microbiol..

[112] Cheng M., Dong J., Lee I.-M., Bottner-Parker K.D., Zhao Y., Davis R.E., Laski P.J., Zhang Z., McBeath J.H. (2014). Group 16SrXII phytoplasma strains, including subgroup 16SrXII-E (‘*Candidatus* Phytoplasma fragariae’) and a new subgroup, 16SrXII-I, are associated with diseased potatoes (*Solanum tuberosum*) in the Yunnan and Inner Mongolia regions of China. Eur. J. Plant Pathol..

[113] Melo L., Silva E., Flôres D., Ventura J., Costa H., Bedendo I. (2013). A phytoplasma representative of a new subgroup, 16SrXIII-E, associated with papaya apical curl necrosis. Eur. J. Plant Pathol..

[114] Fernández F.D., Meneguzzi N.G., Guzmán F.A., Kirschbaum D.S., Conci V.C., Nome C.F., Conci L.R. (2015). Detection and identification of a novel 16SrXIII subgroup phytoplasma associated with strawberry red leaf disease in Argentina. Int. J. Syst. Evol. Microbiol..

[115] Lee I.-M., Bottner K.D., Secor G., Rivera-Varas V. (2006). ‘*Candidatus* Phytoplasma americanum’, a phytoplasma associated with a potato purple top wilt disease complex. Int. J. Syst. Evol. Microbiol..

[116] Kakizawa S., Oshima K., Kuboyama T., Nishigawa H., Jung H.-Y., Sawayanagi T., Tsuchizaki T., Miyata S., Ugaki M., Namba S. (2001). Cloning and expression analysis of phytoplasma protein translocation genes. Mol. Plant Microbe Interact..

[117] Oshima K., Kakizawa S., Nishigawa H., Jung H.-Y., Wei W., Suzuki S., Arashida R., Nakata D., Miyata S., Ugaki M. (2004). Reductive evolution suggested from the complete genome sequence of a plant-pathogenic phytoplasma. Nat. Genet..

[118] Arashida R., Kakizawa S., Hoshi A., Ishii Y., Jung H.-Y., Kagiwada S., Yamaji Y., Oshima K., Namba S. (2008). Heterogeneic dynamics of the structures of multiple gene clusters in two pathogenetically different lines originating from the same phytoplasma. DNA Cell Biol..

[119] Jomantiene R., Davis R.E. (2006). Clusters of diverse genes existing as multiple, sequence-variable mosaics in a phytoplasma genome. FEMS Microbiol. Lett..

[120] Toruño T.Y., Musić M.S., Simi S., Nicolaisen M., Hogenhout S.A. (2010). Phytoplasma PMU1 exists as linear chromosomal and circular extrachromosomal elements and has enhanced expression in insect vectors compared with plant hosts. Mol. Microbiol..

[121] Weintraub P.G., Beanland L. (2006). Insect vectors of phytoplasmas. Annu. Rev. Entomol..

[122] Wei W., Kakizawa S., Suzuki S., Jung H.-Y., Nishigawa H., Miyata S., Oshima K., Ugaki M., Hibi T., Namba S. (2004). In planta dynamic analysis of onion yellows phytoplasma using localized inoculation by insect transmission. Phytopathology.

[123] Bertaccini A., Oshima K., Kakizawa S., Duduk B., Namba S., Brown J.K. (2016). Dissecting the multifaceted mechanisms that drive leafhopper host–phytoplasma specificity. Vector-Mediated Transmission of Plant Pathogens.

[124] Kakizawa S., Oshima K., Ishii Y., Hoshi A., Maejima K., Jung H.-Y., Yamaji Y., Namba S. (2009). Cloning of immunodominant membrane protein genes of phytoplasmas and their in planta expression. FEMS Microbiol. Lett..

[125] Suzuki S., Oshima K., Kakizawa S., Arashida R., Jung H.-Y., Yamaji Y., Nishigawa H., Ugaki M., Namba S. (2006). Interaction between the membrane protein of a pathogen and insect microfilament complex determines insect-vector specificity. Proc. Natl. Acad. Sci. USA.

[126] Galetto L., Bosco D., Balestrini R., Genre A., Fletcher J., Marzachì C. (2011). The major antigenic membrane protein of ‘*Candidatus* Phytoplasma asteris’ selectively interacts with ATP synthase and actin of leafhopper vectors. PLoS ONE.

[127] Boonrod K., Munteanu B., Jarausch B., Jarausch W., Krczal G. (2012). An immunodominant membrane protein (Imp) of ‘*Candidatus* Phytoplasma mali’ binds to plant actin. Mol. Plant Microbe Interact..

[128] Oshima K., Ishii Y., Kakizawa S., Sugawara K., Neriya Y., Himeno M., Minato N., Miura C., Shiraishi T., Yamaji Y. (2011). Dramatic transcriptional changes in an intracellular parasite enable host switching between plant and insect. PLoS ONE.

[129] Oshima K., Kakizawa S., Arashida R., Ishii Y., Hoshi A., Hayashi Y., Kagiwada S., Namba S. (2007). Presence of two glycolytic gene clusters in a severe pathogenic line of ‘*Candidatus* Phytoplasma asteris’. Mol. Plant Pathol..

[130] Himeno M., Kitazawa Y., Yoshida T., Maejima K., Yamaji Y., Oshima K., Namba S. (2014). Purple top symptoms are associated with reduction of leaf cell death in phytoplasma-infected plants. Sci. Rep..

[131] Hoshi A., Oshima K., Kakizawa S., Ishii Y., Ozeki J., Hashimoto M., Komatsu K., Kagiwada S., Yamaji Y., Namba S. (2009). A unique virulence factor for proliferation and dwarfism inplants identified from a phytopathogenic bacterium. Proc. Natl. Acad. Sci. USA.

[132] Sugawara K., Honma Y., Komatsu K., Himeno M., Oshima K., Namba S. (2013). The alteration of plant morphology by small peptides released from the proteolytic processing of the bacterial peptide TENGU. Plant Physiol..

[133] Minato N., Himeno M., Hoshi A., Maejima K., Komatsu K., Takebayashi Y., Kasahara H., Yusa A., Yamaji Y., Oshima K. (2014). The phytoplasmal virulence factor TENGU causes plant sterility by downregulating of the jasmonic acid and auxin pathways. Sci. Rep..

[134] Arashida R., Kakizawa S., Ishii Y., Hoshi A., Jung H.-Y., Kagiwada S., Yamaji Y., Oshima K., Namba S. (2008). Cloning and characterization of the antigenic membrane protein (Amp) gene and in situ detection of Amp from malformed flowers infected with Japanese hydrangea phyllody phytoplasma. Phytopathology.

[135] Himeno M., Neriya Y., Minato N., Miura C., Sugawara K., Ishii Y., Yamaji Y., Kakizawa S., Oshima K., Namba S. (2011). Unique morphological changes in plant pathogenic phytoplasma-infected petunia flowers are related to transcriptional regulation of floral homeotic genes in an organ-specific manner. Plant J..

[136] MacLean A.M., Sugio A., Makarova O.V., Findlay K.C., Grieve V.M., Toth R., Nicolaisen M., Hogenhout S.A. (2011). Phytoplasma effector SAP54 induces indeterminate leaf-like flower development in *Arabidopsis* plants. Plant Physiol..

[137] Maejima K., Iwai R., Himeno M., Komatsu K., Kitazawa Y., Fujita N., Ishikawa K., Fukuoka M., Minato N., Yamaji Y. (2014). Recognition of floral homeotic MADS domain transcription factors by a phytoplasmal effector, phyllogen, induces phyllody. Plant J..

[138] MacLean A.M., Orlovskis Z., Kowitwanich K., Zdziarska A.M., Angenent G.C., Immink R.G., Hogenhout S.A. (2014). Phytoplasma effector SAP54 hijacks plant reproduction by degrading MADS-box proteins and promotes insect colonization in a RAD23-dependent manner. PLoS Biol..

[139] Kitazawa Y., Iwabuchi N., Himeno M., Sasano M., Koinuma H., Nijo T., Tomomitsu T., Yoshida T., Okano Y., Yoshikawa N. (2017). Phytoplasma-conserved phyllogen proteins induce phyllody across the Plantae by degrading floral MADS domain proteins. J. Exp. Bot..

[140] Bertaccini A., Paltrinieri S., Contaldo N. (2019). Standard detection protocol: PCR and RFLP analyses based on 16S rRNA gene. Phytoplasmas.

[141] Bianco P.A., Romanazzi G., Mori N., Myrie W., Bertaccini A., Bertaccini A., Weintraub P.G., Rao G.P., Mori N. (2019). Integrated management of phytoplasma diseases. Phytoplasmas: Plant Pathogenic Bacteria-II Transmission and Management of Phytoplasma Associated Diseases.

[142] Tanno K., Maejima K., Miyazaki A., Koinuma H., Iwabuchi N., Kitazawa Y., Nijo T., Hashimoto M., Yamaji Y., Namba S. (2018). Comprehensive screening of antimicrobials to control phytoplasma diseases using an in vitro plant-phytoplasma co-culture system. Microbiology.

[143] Davies D.L., Clark M.F. (1994). Maintenance of mycoplasma-like organisms occurring in *Pyrus* species by micropropagation and their elimination by tetracycline therapy. Plant Pathol..

[144] Aldaghi M., Massart S., Druart P., Bertaccini A., Jijakli M.H., Lepoivre P. (2008). Preliminary in vitro evaluation of antimicrobial activity of some chemicals and essential oils on apple proliferation disease. Commun. Appl. Biol. Sci. Ghent Univ..

[145] Bertaccini A. (2021). Containment of phytoplasma-associated plant diseases by antibiotics and other antimicrobial molecules. Antibiotics.

